# Improving oxidative stability of cream powder using pomegranate concentrate and peel extract

**DOI:** 10.1002/fsn3.4248

**Published:** 2024-07-17

**Authors:** Seyed Ayuob Khademi, Mohammad Hadi Eskandari, Mohammad Taghi Golmakani, Mehrdad Niakousari, Hadi Hashemi

**Affiliations:** ^1^ Department of Food Science and Technology, School of Agriculture Shiraz University Shiraz Iran

**Keywords:** cream powder, oxidative stability, pomegranate concentrate, pomegranate peel

## Abstract

In this study, the influence of pomegranate peel extract (PPE) and pomegranate concentrate (PC) on improving the oxidative stability of cream powder in comparison with synthetic antioxidant (Butylated hydroxytoluene (BHT)) during the storage period at 20°C (8 weeks) and 45°C (4 weeks) was evaluated. The finding exhibited that peroxide, *p*‐anisidine, and total oxidation (TOTOX) values increased during the storage period. Samples containing PPE and PC showed suitable performance in controlling the cream powder oxidation. Also, PPE showed the highest antioxidant activity and can be proposed as a suitable replacement for a synthetic antioxidant (BHT). L* and b* values increased, while a* value decreased during the storage period. Also, the pH of the powders decreased significantly throughout the storage (reached nearly 6). Microbial counts of cream powders did not change during the storage period. The control sample showed the highest overall acceptability. Antioxidants of pomegranate can be proposed to control the oxidative stability of fat‐rich food products.

## Graphical abstract

In this study, the influence of pomegranate peel extract (PPE) and pomegranate concentrate (PC) was evaluated on improving the oxidative stability of cream powder. The finding exhibited that peroxide, *p*‐anisidine, and TOTOX values increased during the storage period. Samples containing PPE and PC showed suitable performance in controlling the cream powder oxidation. In addition, PPE showed the highest antioxidant activity and can be proposed as a suitable replacement for synthetic antioxidants. Microbial counts of cream powders did not change during the storage period.

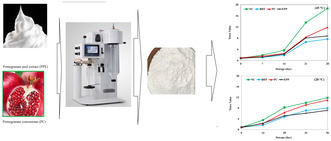

## 1 INTRODUCTION

Different unit operations are applied in the manufacture of full‐cream milk powder. Normally, at first, fat to solids nonfat (SNF) ratio of milk is adjusted, and then other processes, such as preheating, concentrating (~45%–50%), homogenizing, and drying, will be performed (Augustin et al., 2014). One of the main operations in the production of full‐cream milk powder is homogenization, which is suitable for improving the physical characteristics of the powder. This process improved full‐cream milk powder's properties by decreasing the droplet size of the fat globules, changing the interfacial properties of the globule, and reducing the free oil droplet in the powder (Vignolles et al., 2009). Unfortunately, powder with high free‐fat content showed low rehydration and flowability and high oxidation (Augustin et al., 2014). Some of the main detrimental changes in the powdered dairy products are lipid oxidation, browning, crystallization of lactose, and particle caking. Lipid oxidation can reduce the shelf life of the product by changing the sensorial properties (Chudy et al., 2015; Himmetagaoglu & Erbay, 2019).

Lipid oxidation is one of the main reasons for the reduced quality and short shelf life of full fat dairy powders produced by the spray‐drying process during the storage period. Today, antioxidants are introduced as a good strategy to reduce the oxidation of lipids (Asl et al., 2018). Synthetic antioxidants have adverse effects on human health at high concentrations; researches exhibited that the uses of natural antioxidants, instead of synthetic antioxidants, have increased in the last decade (Baldin et al., 2016; Jiang & Xiong, 2016). Also, the demand for green‐labeled food incorporated with natural antioxidants was increased by consumers. Natural antioxidants can be extracted from different fruits, plants, vegetables, oilseeds, spices, and cereals (Yamazaki et al., 2010). Also, the Food and Drug Administration (FDA) recognizes these extracts as Generally Recognized as Safe (GRAS) ingredients for application in food (Nieto et al., 2013). Researchers used natural antioxidants to reduce the oxidation of lipids in various dairy powders. Verma et al. (2023) evaluated the cream powder containing nanocurcumin and reported that nanocurcumin improved the oxidative stability of cream powder. In addition, cream powder containing sodium caseinate showed higher antioxidant activity during 2 months of storage period at 50°C. Milinčić et al. (2022) studied the application of grape–pomace–seed extract in goat milk powder and reported that the natural antioxidant improved antioxidant parameters of the goat milk powder. The total phenolic content (TPC), total antioxidant capacity (TAC), ferric reducing antioxidant power (FRAP), ABTS (2,2‐azino‐bis‐3‐ethylbenzothiazoline‐6‐sulfonic acid) radical scavenging activity, and ferrous ion chelating (FIC) capacity of the sample containing grape–pomace–seed extract were, respectively, 4.5, 4, 16, 6.5, and 2.5 times higher than those of control sample.

The pomegranate (*Punica granatum* L.) was grown in different microclimatic areas. The main countries involved in the production of this fruit are Iran, USA, Turkey, and Egypt (Firuzi et al., 2019). Today, 3 million tons of pomegranate is produced yearly by the main countries. The production of pomegranate was 790,000 tons in 2013 and 1,100,000 tons in 2022 (18% of the total production in the world) in Iran. The increase in the harvesting of this fruit in the world is observed because of improving consumer information on the benefits related to its bioactive ingredients (Fischer et al., 2011). These bioactive ingredients are present in different parts of a pomegranate fruit, and they have various medicinal and functional properties, such as, anticancer, antioxidant, and anti‐atherosclerotic characteristics. The main ingredients of pomegranate are sugars, acids, vitamins, polyphenols, polysaccharides, and minerals. Also, ellagic acid, anthocyanins, tannins, and phytoestrogen flavonoids are the main types of phenolic ingredients (Hadree et al., 2023). Pomegranate juice (PJ) is a popular drink recognized by consumers due to the phenolic ingredients, including ellagic acid, anthocyanins, tannins, and flavonoids (Liu et al., 2013; Oziyci et al., 2013; Viuda‐martos et al., 2013). DPPH (2,2‐diphenyl‐1‐picrylhydrazyl), FRAP, and TPC of pomegranate juice and pomegranate peel powder were reported to be 512 and 1248 μmol TE/g (micromole Trolox equivalent per gram), 87 and 633 μmol TE/g, and 1 and 219 mg GAE/g (milligrams gallic acid equivalent per gram), respectively (Gutiérrez‐Pacheco et al., 2021).

To the best of our knowledge, there was no report on the effect of natural antioxidants including pomegranate peel extract (PPE) and pomegranate concentrate (PC) on cream powder's properties. This study aimed to evaluate the influence of PPE and PC on proteins’ and lipids’ oxidation, physicochemical and sensory characteristics of cream powder against BHT throughout the storage period at 20°C (8 weeks) and 45°C (4 weeks).

## 2 MATERIALS AND METHODS

### 2.1 Materials

Cream (40% fat), milk (1.5% fat), skimmed milk powder, and milk protein concentrate (MPC) were prepared from Pegah Dairy Co. (Shiraz, Iran). All chemicals and culture media were prepared by Merck Co. (Darmstadt, Germany).

### 2.2 Preparation of PPE and PC

The Rabab pomegranates were purchased in October 2014 from Rudkhor village, near the Neyriz city (Fars province, Iran; 28.9680° N, 54.9587° E). After washing the mature pomegranate fruits, they were cut to separate the arils and the peel. PC was produced by filtration of PJ with filter paper (Whatman No. 1) and concentrated by evaporation at 40°C. The peel was resized into small parts and dried by a hot air dryer at 40°C for 2 days. The dried samples were cooled and powdered by a grinder and sieved (60 mesh), packed, and kept at 25°C in high‐density polyethylene (HDPE) bags. Aqueous peel powder solution (4%w/v g) was prepared and boiled for 5 min followed by filtration with filter paper (Whatman No. 1) and concentrated by evaporation at 40°C for the preparation of PPE (Naveena et al., 2008).

#### 2.2.1 Total phenolic content

Total phenolic content (TPC) was determined by the Folin–Ciocalteu technique reported by Shahbazi et al. (2018) with some modifications. PPE and PC (50 mg) were suspended in 100 mL of acetone:distilled water (6:4). Exactly, 200 μL of this solution was added to 1000 μL of aqueous Folin–Ciocalteu (10% w/w) and was stored in the dark condition at 25°C for 15 min. Then, the prepared suspension was mixed with 0.8 mL of 7.5% sodium carbonate solution and kept in the dark ambiance for 60 min. Afterwards, the absorption value of samples was measured at 765 nm and converted to the value of TPC by a gallic acid (GA) standard curve (0.25–2.5 mg/mL). TPC is reported as milligrams (mg) of gallic acid equivalent (GAE) per milliliter (mL) of sample.

### 2.3 Cream powder production

Cream premix was made (50% w/w) by combining skimmed milk powder (200 g), milk (6.6 kg), and cream (3.25 kg) with distilled water at a constant temperature of 33°C. After that, the temperature was raised to 43°C and 75 g milk protein concentrate and 20 g sodium carbonate were added. In this step, antioxidants were added according to Table 1 (the amount of natural antioxidants was replaced with 200 ppm BHT based on TPC). Then, the temperature was increased to 55°C and kept for 60 min and then 60 g sodium caseinate and 50 mL lecithin were added. Then, the temperature was increased to 66°C and kept for 60 min and then 75 g sodium lactose, 40 g sucrose, and 50 g maltodextrin were added. The mixture was homogenized at 100 bar in the first stage and 20 bar in the second stage using a two‐stage homogenizer (APV, Crawley, UK).

**TABLE 1:** Total phenolic content of pomegranate samples.

	Dry matter (mg/mL)	Total phenolic content (μg/mL)	Antioxidant content^a^
Pomegranate peel extract	10.0	0.269	50.00
Pomegranate concentrate	16.8	0.211	68.96

^a^ mL equal to 200 mg BHT.

The samples were pumped and dried in a Maham Sanat spray dryer (Neyshabur, Iran) equipped with a nozzle with an orifice of 1.5 mm diameter. The samples were pumped to the dryer chamber with 1000 mL/h flow rate. Also, our samples were dried at inlet and outlet air temperatures of 160 and 60°C, respectively. The fine dried samples were granulated and removed from the cyclone (the so‐called dusty) at the top of the drying chamber. The powders were collected in polyethylene (PE) bags and kept at 20°C (for 8 weeks) and 45°C (for 4 weeks).

#### 2.3.1 Chemical composition

Moisture, protein, fat, lactose, and ash contents of the cream powders were determined based on the methods reported by Almanza‐Rubio et al. (2016).

#### 2.3.2 Peroxide, *p*‐anisidine, and TOTOX values

Lipid extraction of cream powder was performed and utilized for measuring the peroxide value (PV), *p*‐anisidine value (AV), and TOTOX value (TV) as lipid indicators. Both, PV and AV, were measured according to Keramat et al. (2016).

#### 2.3.3 Fatty acid profile

To study the fatty acid profile, a gas chromatography (GC) system (Beiten Inc., China) equipped with a BPX70 GC column (30 m long, 0.25 μm internal diameter, and 0.25 μm film thickness) and flame ionization detector (FID) were applied. At first, 0.2 g extracted oil was added to 10 mL acetyl chloride (5% in methanol) and placed in the oven (85°C) for 65 min. After that, 5 mL double‐distilled water (DDW) was added, homogenized for 5 min, and centrifuged in 4000 *g* for 5 min at 25°C. The column temperature was increased from 80°C to 200°C (15°C per minute) and kept at 200°C for 10 min. Then, it reached 220°C by increasing 20°C per minute and was kept at 220°C for 5 min. The temperature of both the injection valve and the detector was 250°C. Nitrogen was used as a carrier gas at a flow rate of 1000 μL/min. Injection to the GC system was conducted using split mode at a ratio of 1:10 (Toorani et al., 2020).

#### 2.3.4 Color analysis

The color of all powder samples was measured by the digital image‐based colorimetric method. Under the same conditions, the picture was taken from the surface powders by a digital camera (Canon, Tokyo, Japan). Image parameters, such as contrast, resolution, and lightness, were set to 62%, 300 dots per inch (dpi), and 62%, respectively. The format of the photograph was JPEG (Joint Photographic Experts Group) and the photo color was analyzed by Adobe Photoshop CS5. Color parameters including L*, a*, and b* were measured for each picture (Golmakani et al., 2021).

#### 2.3.5 pH

The pH value was measured by the standard approach reported by Bemer et al. (2016). In addition, pH was measured after mixing 10 g of powder in 100 mL of distilled water with a digital pH meter (Starter 3000, Ohaus, Nänikon, Switzerland).

#### 2.3.6 Total count

Total aerobic bacteria of the samples were quantified every 2 weeks using the method described by Ismail et al. (2023).

#### 2.3.7 Sensory evaluation

At least 20‐member trained panelists from the Department of Food Science and Technology of Shiraz University (Shiraz, Iran) performed the sensory evaluation. This test was performed based on a 5‐point hedonic scale technique (1 = very bad to 5 = very good). Samples for sensorial evaluation were coded and evaluated by trained panelists for acceptance rate of aroma, taste, color, texture, and overall acceptance value. For this purpose, the powders were analyzed after 1 week of storage at 4°C (Bemer et al., 2016).

### 2.4 Statistical analysis

All tests were performed in a completely randomized design form and repeated at least three times. Duncan's multiple range test was applied to compare the mean values when the effect was significant (*p* < .05). The data are reported in the form of mean and standard deviation (Gahruie et al., 2017). SAS software was used for statistical analysis (V.9.1, Cary, NC, USA).

## 3 RESULTS AND DISCUSSION

### 3.1 Chemical composition of cream powder

Cream powder contains 3.46% moisture, 53.98% lipid, 14.65% protein, 23.26% carbohydrate, and 4.54% ash.

### 3.2 Oxidative stability of cream powder

#### 3.2.1 PV, AV, and TV

**FIGURE 1:**
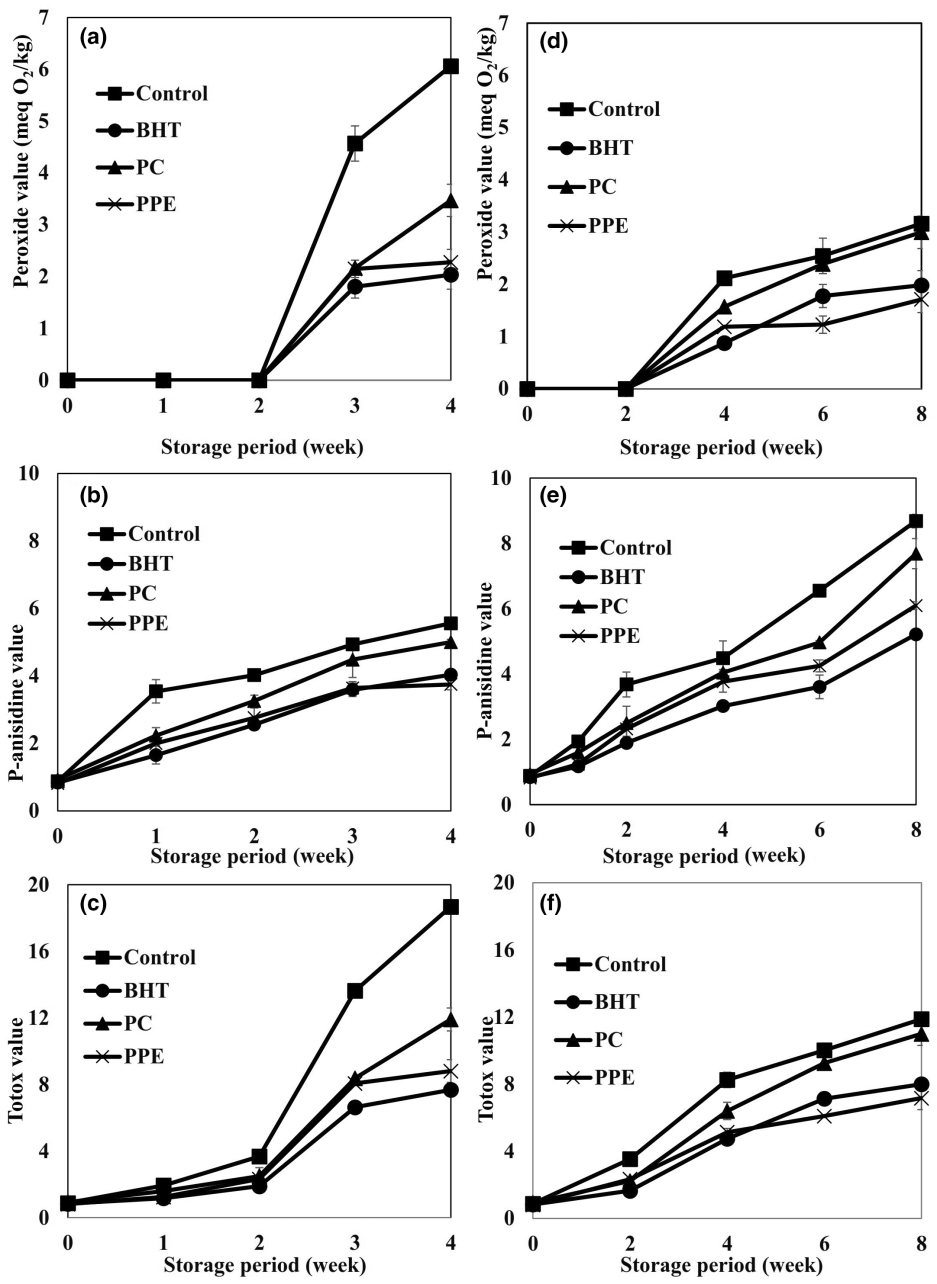
Effects of pomegranate peel extract (PPE), pomegranate concentrate (PC), and BHT addition on peroxide, *p*‐anisidine, and TOTOX values of cream powder samples during the storage period at 20°C (a–c) and 45°C (d–f).

Effects of natural (PPE and PC) and synthetic (BHT) antioxidants on PV of cream powder samples are shown in Figure 1a,d. The increasing rate of PV for control was higher than powders containing both types of antioxidants. The PVs for control arrived at 6.06 ± 0.07 and 3.16 ± 0.15 meq O_2_/kg after storage at 45°C (4 weeks) and 20°C (8 weeks), respectively. The PVs of samples containing PPE showed similar increasing trends to that of BHT. The PVs of PPE, PC, BHT, and control samples increased to 2.27, 3.47, 2.03, and 6.06 after 4 weeks and to 1.71, 2.99, 1.98, and 3.16 after 8 weeks, respectively. The findings represented that the storage temperature had sharp effects on the PV of the sample, especially the control and PC sample. Similar results on PV increase by increasing the storage temperature were reported by Lloyd et al. (2009) on the whole milk powder.

The unsaturated and saturated carbonyl compounds of oils with high molecular weights were analyzed by anisidine test (Frankel, 2014). Effects of natural (PPE and PC) and synthetic (BHT) antioxidants on the AV of cream powder samples stored at different temperatures are shown in Figure 1b,e. The AVs of all samples raised during the storage period, while the increasing rate at 45°C was higher than that at 20°C. The fact could be related to the higher hydroperoxide decomposition rate than its formation rate. At the end of the storage period (8 weeks at 20°C), the AVs of BHT, PC, PPE, and control sample reached 5.22 ± 0.15, 7.69 ± 0.46, and 6.10 ± 0.12, and 8.69 ± 0.21 mg/kg, respectively, while, after storage for 4 weeks at 45°C, the AVs of BHT, PC, PPE, and control sample reached 4.04 ± 0.12, 5.01 ± 0.08, 3.75 ± 0.16, and 5.56 ± 0.11 mg/kg, respectively.

The TVs determine both primary and secondary oxidation products during the oxidation of lipids. The addition of PPE, PC, and BHT caused significant reduction in TVs of cream powders both stored at 20 and 45°C compared to the control. The TVs of BHT and PPE samples did not have any significant differences (*p* < .05). This finding represented the fact that the delay in production of both oxidation products of cream powder in PPE and BHT sample was comparable. Therefore, PPE is a good source of bioactive ingredients, which can retard the oxidation products of cream powder.

### 3.3 Fatty acid profile

Effects of natural (PPE and PC) and synthetic antioxidants (BHT) on the fatty acid profile of cream powder samples are shown in Table 2. The main fatty acids of cream powder samples were palmitic (36%), oleic (24%), lauric (12%), and stearic acids (9%), respectively. This profile is inconsistent with those reported by Liu et al. (2016). Also, Abarghuei et al. (2014) reported that C14:0, C16:0, C18:0, and C18:1 were the main fatty acids of milk powder. These differences can be noticed because of the differences in feeding, climate conditions, and extraction methods (Mele et al., 2016). The fatty acid profiles of cream powders after PPE, PC, or BHT addition and also after storage at 20°C (8 weeks) and 45°C (4 weeks) (Table 2) have not shown any significant differences. Himmetagaoglu and Erbay (2019) evaluated the influence of spray‐drying process on the free fatty acid content of microencapsulated cream powder. They reported that palmitic, oleic, and stearic acids were the main free fatty acids, respectively. The fatty acid composition of milk is usually related to the fatty acids of the diet and de novo synthesis. Short‐chain fatty acids (C4:0–C14:0) and some C16:0 are synthesized by a de novo method, while the remaining C16:0 and the long‐chain fatty acids are affected by the dietary fats. So, the bovine diet has the main influence on the fatty acid profile of milk powder (Clarke et al., 2020).

**TABLE 2:** Effect of pomegranate peel extract (PPE), pomegranate concentrate (PC), and BHT addition on fatty acids profile of cream powder during storage at different temperatures (°C).

No.	Fatty acid	Control		BHT		PC^a^		PPE	
		20ºC^a^	45ºC	20ºC	45ºC	20ºC	45ºC	20ºC	45ºC
1	C4:0	2.58	3.47	2.22	1.62	5.38	3.81	2.46	3.51
2	C6:0	1.16	1.37	1.62	1.45	2.31	1.53	1.83	1.52
3	C8:0	1.42	1.43	1.25	1.31	1.22	2.57	1.31	1.35
4	C10:0	3.48	3.08	3.01	3.70	3.03	3.97	3.23	4.50
5	C12:0	3.39	8.46	3.39	3.44	3.13	3.93	3.66	3.82
6	C14:0	11.64	10.12	10,82	12.27	11.54	14.25	14.15	12.23
7	C16:0	35.80	33.65	35.16	33.75	35.63	33.38	34.77	33.50
8	C18:0	9.35	8.53	9.02	7.23	8.67	8.14	8.67	9.13
9	C18:1(9)	23.92	20.33	20.52	23.66	22.05	21.42	21.85	22.80
10	C18:2(6)	5.46	5.64	6.84	6.74	6.62	4.79	6.23	6.70
11	C18:3(3)	1.80	3.93	3.11	3.77	3.43	2.21	1.84	0.94
12	Saturated	68.82	70.11	66.54	65.83	67.90	71.58	70.08	69.56
13	Unsaturated	31.18	29.89	33.46	34.17	32.10	28.42	29.92	30.44
14	Polyunsaturated	7.26	9.57	9.95	10.51	10.05	7.00	8.07	7.66

^a^ 20°C for 8 weeks and 45°C for 4 weeks.

### 3.4 Color analysis

**FIGURE 2:**
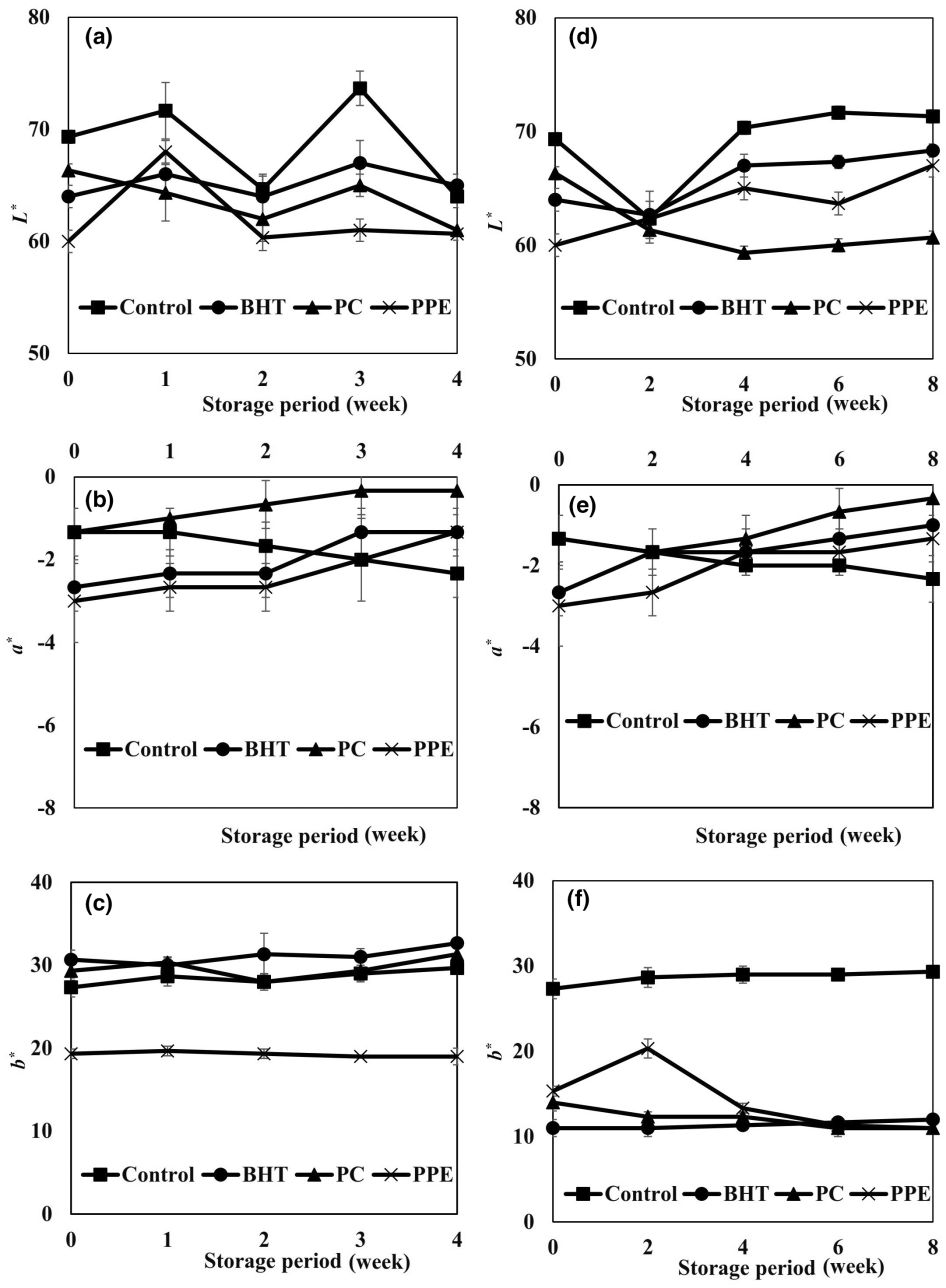
Effects of pomegranate peel extract (PPE), pomegranate concentrate (PC), and BHT addition on L*, a*, and b* values of cream powder samples during the storage period at 20°C (a–c) and 45°C (d–f).

Effects of PPE, PC, and BHT antioxidants on L*, a*, and b* values of cream powder samples are shown in Figure 2. A significant decrease in L* value was shown between the samples incorporated with antioxidants at the beginning of the storage period in comparison with the control. During the storage period at 20°C, L* values increased significantly (*p* < .05), but in powders kept at 45°C, L* values of control and PC samples decreased significantly, while L* values of BHT and PPE samples remained constant. The color, appearance, and flavor of milk powder changed after the starting of the Maillard reactions. Usually, the nonenzymatic browning of powders was determined using the color of the powder and reconstituted powder surface. Tang and Chen (2000) evaluated the effects of dark (4, 25, and 45°C) and light (25°C) storage on the stability of the pigment in freeze‐dried powders. They reported that the L* values were reduced during the storage period and by raising the temperature. Also, low L* values could be due to the creation of brown pigments in the carbohydrate–protein interaction because of the Maillard reactions (Grigioni et al., 2007).

Pomegranate peel extract samples showed the lowest values of a* at the beginning of the storage. The b* value was reduced significantly at the beginning of the storage in the powders fortified with antioxidants compared to the control sample. The b* values of PC and PPE samples reduced significantly through the storage at 25°C, but in the powders kept at 45°C, b* values remained constant. This is due to the nonenzymatic browning, as determined from the cream powder color, and it was increased by increasing the storage temperature, and based on the findings of Jose et al. (2023), they reported a significant increase in Maillard reaction products at storage temperatures higher than 30°C. Also, Phosanam et al. (2020) found changes in Maillard reaction products in the powders stored at 35°C. These reports showed that temperature of storage is one of the main reasons for the Millard reaction. The cream powder composition and its production process are important factors affecting the color of the cream powder samples.

### 3.5 pH

**FIGURE 3:**
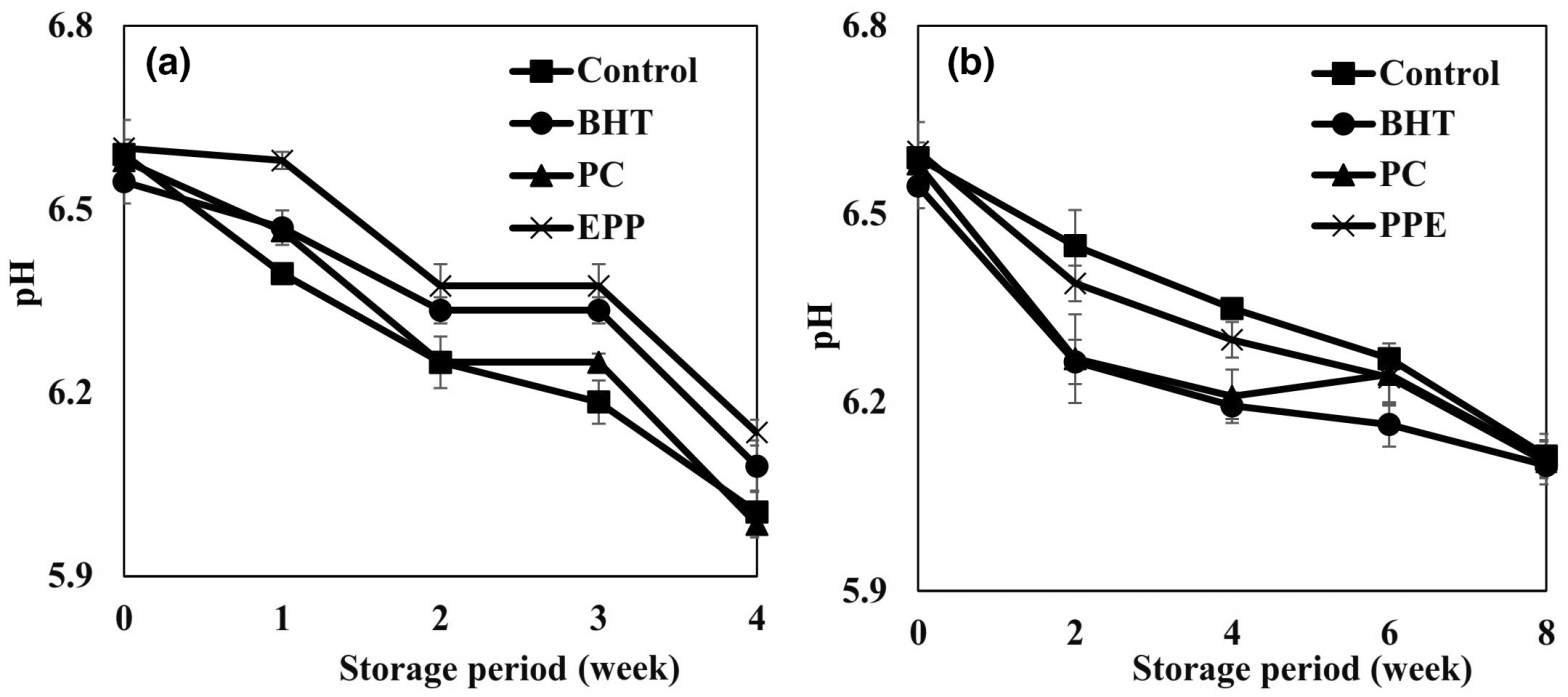
Effects of pomegranate peel extract (PPE), pomegranate concentrate (PC), and BHT addition on pH of cream powder samples during the storage period at 20°C (a) and 45°C (b).

Effect of natural and synthetic antioxidants on the pH of cream powder samples is reported in Figure 3. All powders showed the same pH values after preparation, ranging from 6.54 to 6.60. The addition of natural and synthetic antioxidants into the cream powders had no significant effects on the pH values during the storage at 25°C (8 weeks) (*p* < .05). However, the lowest pH values are related to the control (6.01) and PC (5.98) samples after the 4 weeks of storage at 45°C, while PPE showed the highest pH value (6.13). pH reduction due to the oxidation of lipids in cream powder and production of free fatty acids was observed (Hashemi Gahruie et al., 2017; Khademi et al., 2022). Due to the low water activity, the bacterial count had no significant effect on the pH of the samples.

### 3.6 Total count

**FIGURE 4:**
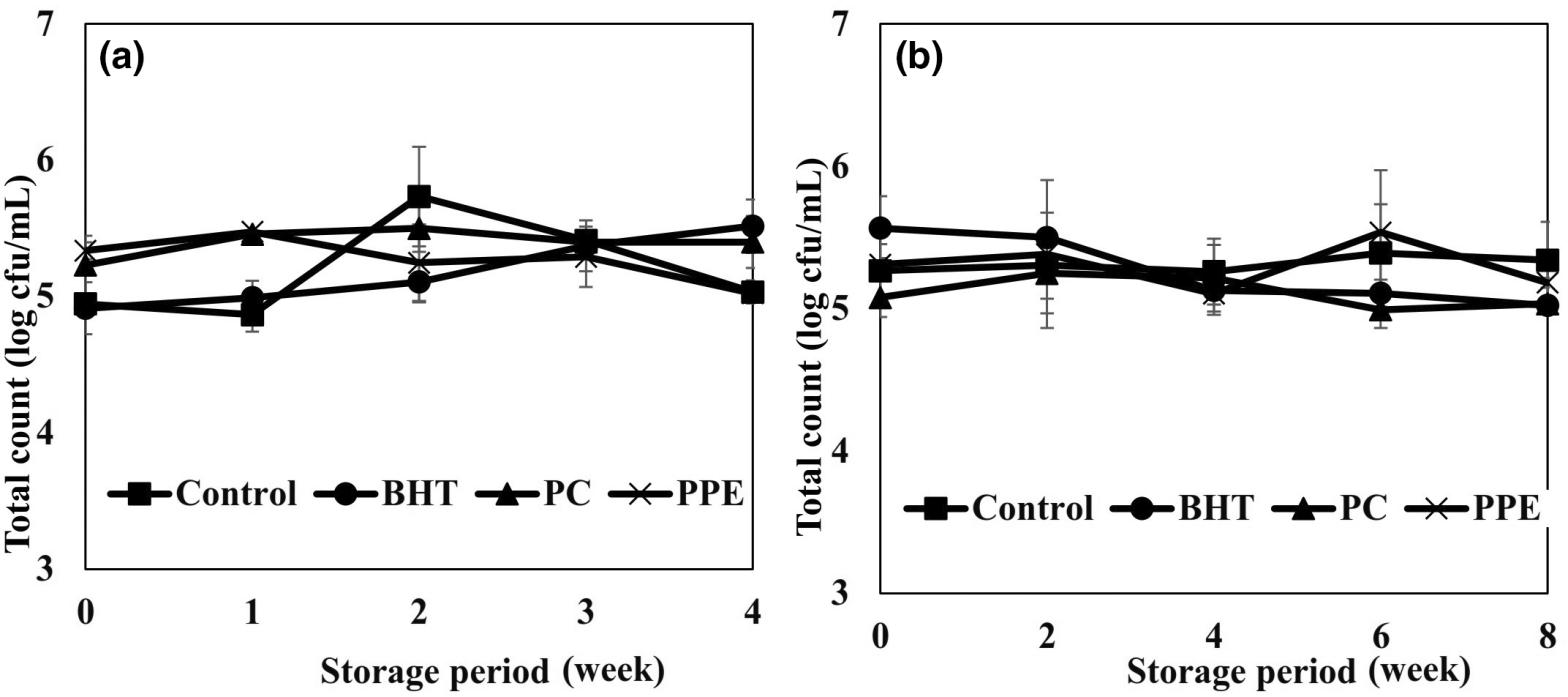
Effects of pomegranate peel extract (PPE), pomegranate concentrate (PC), and BHT addition on total count (log CFU/mL) of cream powder samples during the storage period at 20°C (a) and 45°C (b).

Microorganisms can decrease the safety and quality of the milk, and usually originate from processing, supply chain, milk collection, and milk production. The microorganisms of raw milk and different processing are two main factors affecting the dairy powder microbiota (McHugh et al., 2020). One of the most important properties based on international standard (ISO 16297:2020) for dairy powder is the total count. The effect of natural and synthetic antioxidants on the total count (log CFU (colony‐forming unit)/mL) of cream powder samples is shown in Figure 4. Total counts of cream powder samples including PPE, PC, BHT, and control were similar at 25°C for 8 weeks (5.04 to 5.34 log CFU/g) and at 45°C for 4 weeks (5.02 to 5.51 log CFU/g). The total count of cream powder sample is usually constant because of its low water activity. Arepally and Goswami (2019) and Tapia et al. (2020) reported constant bacterial count in the spray‐dried particle. Pramularsih et al. (2022) studied the total bacterial count in different dairy powders. They reported that *Bacillus licheniformis* with 52%, *Anoxybacillus flavithermus* with 31%, *Geobacillus stearothermophilus* with 15%, and *Bacillus pumilus* with 2% were the main isolated bacteria from different powder samples.

### 3.7 Sensory evaluation

**FIGURE 5:**
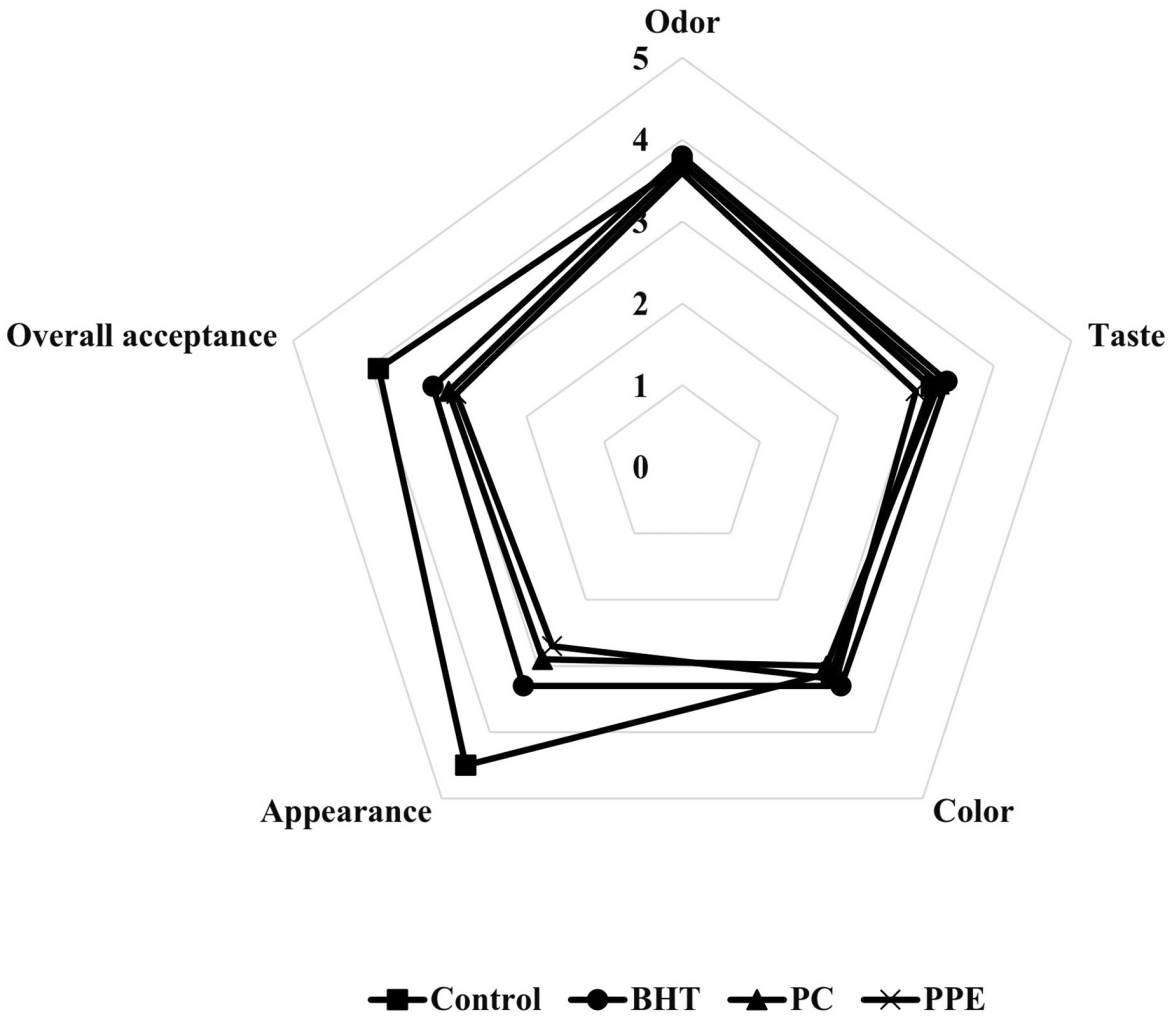
Effects of pomegranate peel extract (PPE), pomegranate concentrate (PC), and BHT addition on sensory properties of cream powder samples after 1‐week storage at 4°C.

Color, odor, and textural properties are the main sensory characteristics, which effect the acceptability of dairy powders by consumers (Gahruie et al., 2015). Effects of natural and synthetic antioxidants on the sensorial properties of cream powder samples are shown in Figure 5. Sensory characteristics, such as color, appearance, taste, odor, and overall acceptance of samples, were assessed and the results represented no significant differences between the samples, except the control with a higher score in terms of appearance and overall acceptance. Usually consumers know cream powder as a food product without additive. In addition, this type of natural antioxidant, due to its special color, odor, and taste, had a significant effect on the sensorial characteristics of the food. These terms are the main reasons for reducing acceptance of product. The effects of pomegranate byproduct on the sensory properties of food products were reported by Kaderides et al. (2021) and Sharayei et al. (2020). They reported that the pomegranate byproduct can change the acceptance of sensorial parameters, such as aroma, taste, color, and general acceptance of the final product. Verma et al. (2022) studied the nanocurcumin fortified milk cream powder through microfluidization and spray‐drying. They reported that although the fortified sample had different sensorial properties due to the curcumin, their sample as a new product had good acceptability by consumers. The odor threshold of most volatile ingredients was higher in fat matrices when compared to the aqueous and air, usually because of the complexity and binding of the matrix. Also, pentanal, heptanal, and hexanal were the main ingredients in the lipid oxidation of milk during the storage (Clarke et al., 2020).

## 4 CONCLUSION

This research aimed to study the feasibility of the replacement of BHT as a synthetic antioxidant with natural antioxidants (PPE and PC) in cream powder formulation, in terms of lipid oxidation, physicochemical, microbial, and sensorial properties of the samples during the storage period at different temperatures. PPE and PC inhibited the lipid oxidation in formulated cream powder better than BHT. This effect was validated by the fatty acid analysis of control and samples containing antioxidants. In addition, PC improved the sensory attributes and microbiological properties of cream powder. The addition of natural and synthetic antioxidants had no significant influence on the pH values of cream powders during the storage period. Therefore, it can be concluded that PPE and PC can be proposed as safe substitutes for BHT in cream powder to improve its oxidative stability. In addition, the main implication of this research is starting to develop green‐labeled dairy powder products.

## AUTHOR CONTRIBUTIONS

**Seyed Ayuob Khademi:** Data curation (equal); formal analysis (equal); methodology (equal); software (equal); writing – original draft (equal). **Mohammad Hadi Eskandari:** Conceptualization (equal); funding acquisition (equal); project administration (equal); resources (equal); supervision (equal); validation (equal). **Mohammad Taghi Golmakani:** Conceptualization (equal); funding acquisition (equal); project administration (equal); resources (equal); supervision (equal); validation (equal); writing – review and editing (equal). **Mehrdad Niakousari:** Data curation (equal); methodology (equal); validation (equal). **Hadi Hashemi:** Data curation (equal); investigation (equal); software (equal); validation (equal); writing – original draft (equal).

## FUNDING INFORMATION

This research project was financially supported by Shiraz University.

## CONFLICT OF INTEREST STATEMENT

The authors declare no conflict of interest.

## Supporting information


Figure S1.‐S2.

## Data Availability

Data available in article supplementary material (Figures S1 and S2).
