# Supplementary material for: Improving oxidative stability of cream powder using pomegranate concentrate and peel extract
Source: Food Sci Nutr. 2024 Jul 17;12(10):7223–32. doi: 10.1002/fsn3.4248 (PMC11521696; doi:10.1002/fsn3.4248)
Supplement: Supplementary file 1 — Figure S1.‐S2. [file FSN3-12-7223-s001.docx]

Fig. S1. Standard curve of gallic acid.

| Control 20 °C | Control 45 °C |
| --- | --- |
| 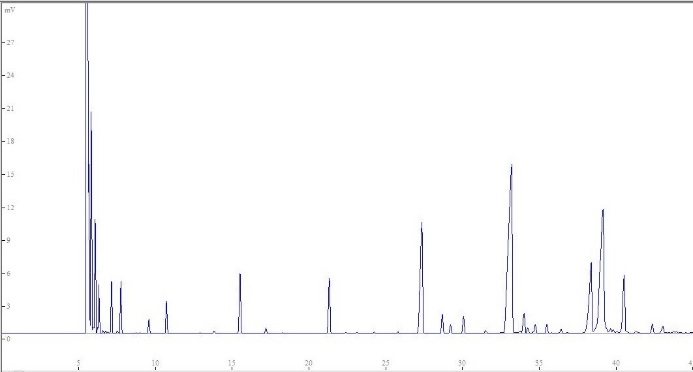 | 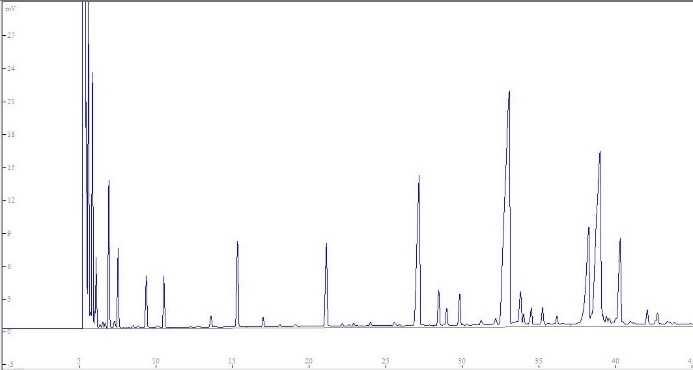 |
| BHT 20 °C | BHT 45 °C |
| 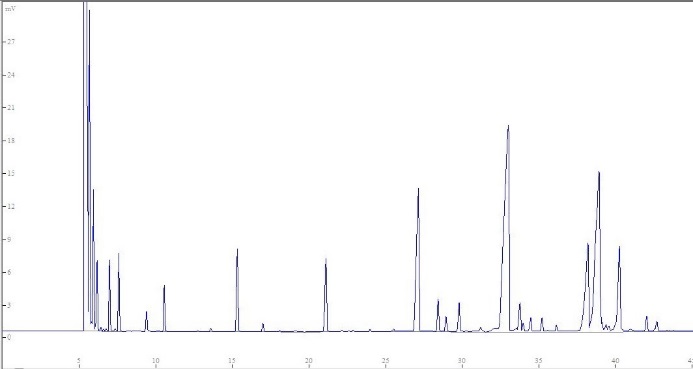 | 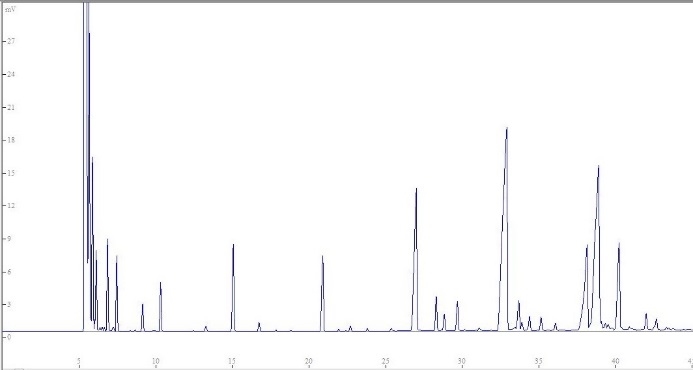 |
| Pomegranate concentrate 20 °C | Pomegranate concentrate 45 °C |
| 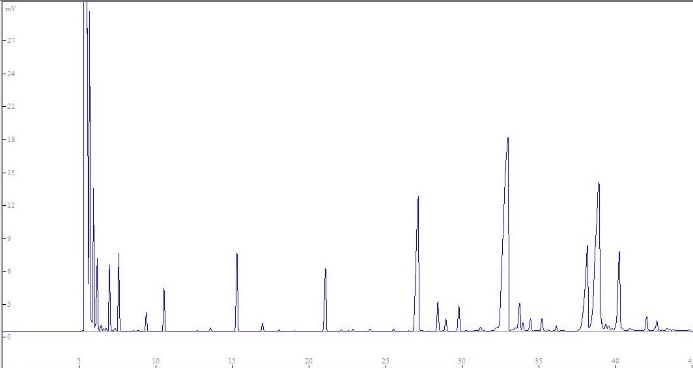 | 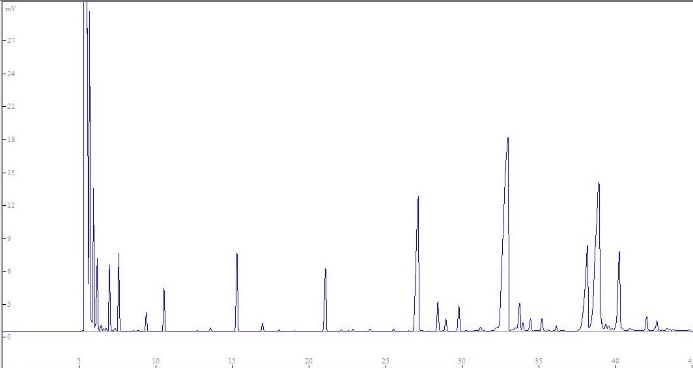 |
| Pomegranate peel extract 20 °C | Pomegranate peel extract 45 °C |
| 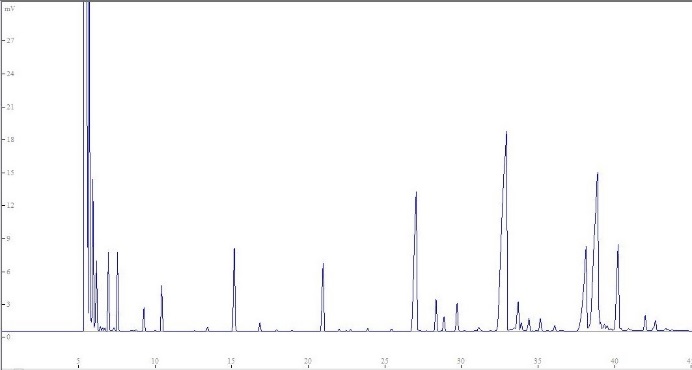 | 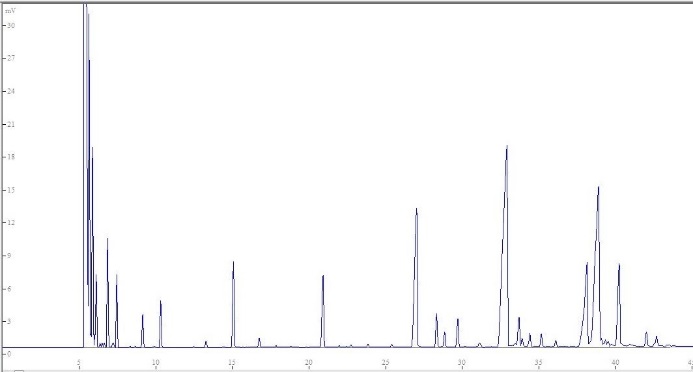 |

Fig S2. GC/FID chromatograms of different cream powder samples.
